# Substrate-Specific Gene Expression in *Batrachochytrium dendrobatidis,* the Chytrid Pathogen of Amphibians

**DOI:** 10.1371/journal.pone.0049924

**Published:** 2012-11-20

**Authors:** Erica Bree Rosenblum, Thomas J. Poorten, Suzanne Joneson, Matthew Settles

**Affiliations:** 1 Department of Environmental Science Policy and Management, University of California, Berkeley, California, United States of America; 2 Initiative for Bioinformatics and Evolutionary Studies, University of Idaho, Moscow, Idaho, United States of America; Imperial College Faculty of Medicine, United Kingdom

## Abstract

Determining the mechanisms of host-pathogen interaction is critical for understanding and mitigating infectious disease. Mechanisms of fungal pathogenicity are of particular interest given the recent outbreaks of fungal diseases in wildlife populations. Our study focuses on *Batrachochytrium dendrobatidis* (Bd), the chytrid pathogen responsible for amphibian declines around the world. Previous studies have hypothesized a role for several specific families of secreted proteases as pathogenicity factors in Bd, but the expression of these genes has only been evaluated in laboratory growth conditions. Here we conduct a genome-wide study of Bd gene expression under two different nutrient conditions. We compare Bd gene expression profiles in standard laboratory growth media and in pulverized host tissue (i.e., frog skin). A large proportion of genes in the Bd genome show increased expression when grown in host tissue, indicating the importance of studying pathogens on host substrate. A number of gene classes show particularly high levels of expression in host tissue, including three families of secreted proteases (metallo-, serine- and aspartyl-proteases), adhesion genes, lipase-3 encoding genes, and a group of phylogenetically unusual crinkler-like effectors. We discuss the roles of these different genes as putative pathogenicity factors and discuss what they can teach us about Bd’s metabolic targets, host invasion, and pathogenesis.

## Introduction

Elucidating the specific mechanisms that pathogens employ to attack their hosts is critical for understanding disease dynamics and pathogen evolution. Pathogens can impact their hosts on many levels, from disrupting organismal physiology to altering specific cellular processes. Pathogens interact with their hosts at the molecular level by secreting and/or presenting proteins that are involved in processes such as host entry, toxicity, immune evasion, and resource acquisition (e.g., [1]–[5]). These pathogenicity factors are encoded and regulated at the molecular level by specific genes and transcription factors (e.g., [6]–[7]). Therefore studies of gene expression can shed light on the molecular changes that affect the production of proteins involved in host invasion. Understanding the molecular mechanisms of pathogenesis can also lead to breakthroughs in disease treatment (e.g., [8]–[9]). However, the mechanisms of interaction between many deadly pathogens and their hosts remain elusive, particularly for emerging pathogens of vertebrates in the wild.

The fungus *Batrachochytrium dendrobatidis* (Bd) is a deadly intracellular pathogen that attacks amphibian skin [10]–[11]. Skin is a particularly sensitive organ in amphibians and plays a critical role in osmoregulation, electrolyte balance and immunity [12]–[13]. Bd infection compromises the integrity of amphibian skin [14]–[15], and the physiological consequences of Bd infection are fatal in many species [16]–[18]. The impact of Bd on amphibians is also dramatic at a global scale. Hundreds of amphibian species around the world are infected with Bd, and the resulting disease - chytridiomycosis - is a major driver of amphibian declines worldwide [19].

There are a number of outstanding questions about the mechanisms of Bd colonization and infection of its amphibian hosts, particularly at the molecular level. For example, we have a limited understanding of the mechanisms of Bd attachment to and invasion of host epidermal cells. Detailed studies of Bd growth and development have been conducted at morphological and ultrastructural levels (e.g., [10]–[11], [20]–[21]), but the molecular and cellular mechanisms of invasion remain to be determined. Additionally, we have a limited understanding of the specific host proteins Bd metabolizes. Bd infects keratinized amphibian tissue [22], and zoospores exhibit positive chemotaxis toward keratin [23]. However, Bd extracellular proteases have not been found to degrade keratin in the lab [24], and there is no direct evidence that Bd metabolizes the keratin of live hosts. Identifying Bd’s metabolic targets is critical for understanding how Bd disrupts the integrity of amphibian skin. Finally, we have a limited understanding of mechanisms of interaction between Bd and the immune system of its hosts. There is some evidence that Bd may be capable of host immune evasion and/or suppression [15], [25], but this hypothesis requires rigorous testing. Identifying the Bd-encoded factors that are involved in disrupting host processes is a powerful resource for devising effective treatments to chytridiomycosis.

Interactions between Bd and amphibian skin are likely mediated by secreted proteases. Proteases are a broad class of proteins that are involved in degrading other peptides. As such, pathogen proteases are responsible for degrading host tissue in many systems (e.g., [26]–[28]). *Secreted* proteases are of particular interest because these molecules can interact directly with host cells, function as pathogenicity factors, and provide clues about a pathogen’s metabolic targets (e.g., [29]–[30]). There are a large number of secreted proteases encoded in the Bd genome, and we have previously proposed several specific families of secreted proteases as putative Bd pathogenicity factors. Specifically, we showed Bd-specific gene family expansions in metallo-, serine-, and aspartyl-protease [31]–[32], gene families thought to be involved in pathogenesis of other fungal pathogens (e.g., [33]–[35]).

Here we evaluate the molecular profile of Bd when it is grown in host tissue. Because we are interested here in host tissue as a nutrient substrate, we grew Bd in sterilized, pulverized host tissue. Using sterilized frog skin instead of live frogs also removes any effect of host immune response or other cutaneous microbes on Bd gene expression. We compare the whole genome expression profile of Bd grown in amphibian skin with that of Bd grown in non-host nutrient conditions. We evaluate whether genes encoding secreted proteases show increased expression in the presence of host tissue, and we identify other genes that may be involved in the host invasion processes. We argue that secreted proteases may serve as pathogenicity factors by allowing Bd to penetrate host cells, metabolize host tissue, and disrupt amphibian skin function.

## Results

**Figure 1:**
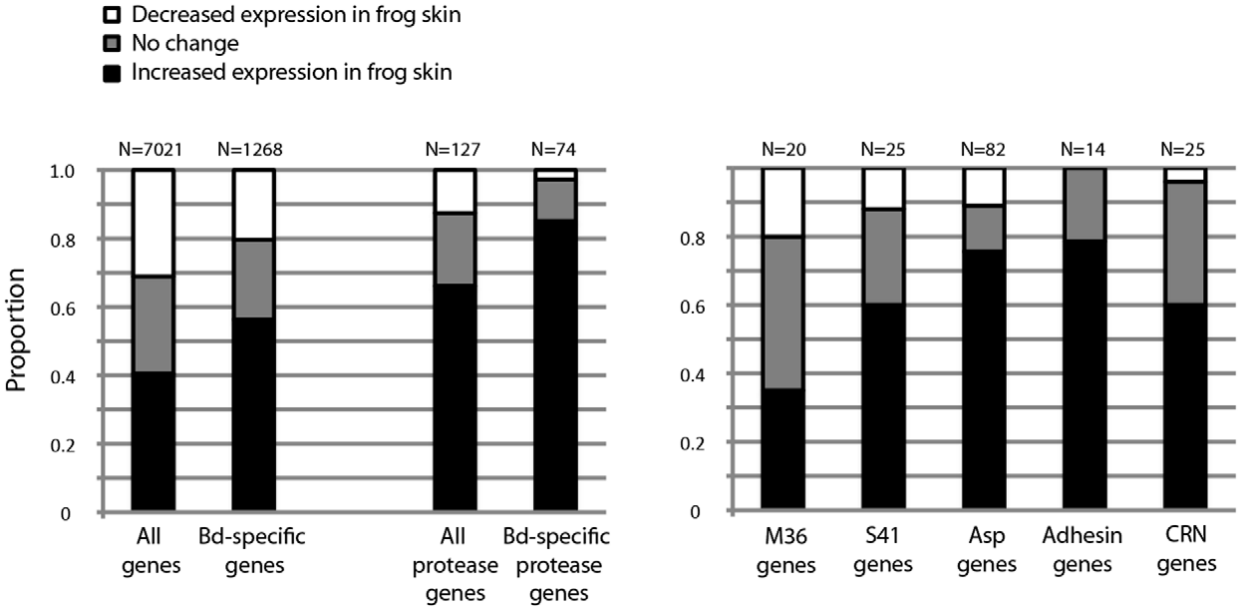
The proportion of Bd genes with significantly increased or decreased expression in the frog skin treatment (relative to the tryptone treatment) shown for all genes, Bd-specific genes, and several families of putative pathogenicity genes. Genes with significant differences in expression were identified using a correction for multiple testing. Abbreviations include M36 = fungalysin metalloprotease, S41 = serine protease, Asp = aspartyl protease, CRN = Crinkler-like effectors.

A large number of genes were differentially expressed when comparing Bd grown on frog skin to Bd grown in tryptone broth (Fig. 1). Of the 7019 total transcripts in the filtered dataset, 5031 (72%) were significantly differentially expressed. A larger proportion of differentially expressed transcripts showed increased expression on frog skin relative to standard growth media (2845/5031, 57%) than decreased expression (2186/5031, 43%). Of the 1268 “Bd-specific” transcripts (those genes identified only in Bd [32]) represented on our array, 973 (77%) were differentially expressed in the two growth conditions. For Bd-specific genes, the proportion of genes showing increased expression in frog skin was quite large (714/973, 73%) relative to the proportion showing decreased expression (259/973, 27%).

Gene set enrichment analyses (GSEA) revealed several GO terms that were overrepresented in the differentially expressed data set (Table 1). We first conducted GSEA for the entire collection of Bd genes represented on our microarray. In this analysis, GO terms significantly overrepresented in the gene set with increased expression in frog skin were “proteolysis” (p = 0.02), and “aspartic-type endopeptidase activity” (p = 0.007). There was also a non-significant enrichment trend for “chitin catabolic process” (p = 0.059) and “chitinase activity” (p = 0.059). GO terms overrepresented in the gene set with increased expression in the tryptone treatment included genes involved in basic cellular function such as cell redox homeostasis (p = 0.03) and endopeptidase activity (p = 0.025). We also conducted GSEA for the subset of “Bd-specific” genes, those genes identified only in Bd [31]. The results for the Bd-specific GSEA were concordant with the results from the full gene set (Table 1) with significant enrichment for proteolysis (p = 0.025), and “aspartic-type endopeptidase activity” (p = 0.025) in the gene set with increased expression in frog skin. Notably, for the Bd-specific gene set, there were no GO categories with significant enrichment for the gene set with decreased expression in frog skin.

**Table 1:** The enrichment of Gene Ontology (GO) categories for the frog skin and tryptone treatments.

	*GO gene set ID*	*Gene set size*	*GO gene set description*	*Normalized enrichment score*	*p-value*
**Biological Process**					
	frog skin				
	GO:0006508	207	proteolysis	1.21	0.020
	GO:0006032	11	chitin catabolic process	1.38	0.059
	tryptone				
	GO:0045454	14	cell redox homeostasis	−1.40	0.030
**Molecular Function**					
	frog skin				
	GO:0004190	77	aspartic-type endopeptidase activity	1.16	0.007
	GO:0004568	11	chitinase activity	1.38	0.059
	tryptone				
	GO:0004175	11	endopeptidase activity	−1.34	0.025
	GO:0004298	11	threonine-type endopeptidase activity	−1.36	0.049
	GO:0008415	16	acyltransferase activity	−1.30	0.049
**Cellular Component**					
	frog skin				
	none				
	tryptone				
	GO:0005839	11	proteasome core complex	−1.36	0.049

The three expanded protease gene families (metallo-, serine-, and aspartyl-proteases) showed striking patterns of increased expression in the frog skin treatment (Fig. 1). Considering all three families of proteases together, 84/127 (66%) transcripts show increased expression on frog skin and 16/127 (13%) transcripts show decreased expression. Considering only the Bd-specific members of these gene families, 63/74 (85%) show increased expression while only 2/74 (3%) show decreased expression. Evaluating each protease family individually, there were a number of genes that showed increased expression in frog skin, were Bd-specific, and showed life stage specific expression patterns (Table 2).

**Table 2:** Selected gene classes with differential expression in the two nutrient treatments.

	*Direction*	*A*	*Log2 fold change*	*Fold change*	*Adjusted* *p-value*	*JGI ID*	*Bd specific*	*Life stage*
**M36**								
	Increased	10.0	1.4	2.6	5.5E−03	BATDEDRAFT_11205		
	Increased	9.3	4.2	18.4	2.0E−05	BATDEDRAFT_1502		S
	Increased	9.0	2.8	7.0	2.0E−06	BATDEDRAFT_16613	*	
	Increased	8.8	2.2	4.6	1.4E−02	BATDEDRAFT_1489		
	Increased	8.6	3.1	8.6	1.1E−04	BATDEDRAFT_1469		S
	Increased	9.3	3.8	13.9	1.2E−06	BATDEDRAFT_12637		S
	Increased	11.7	1.4	2.6	1.9E−02	BATDEDRAFT_1639		S
	Decreased	11.9	−2.6	6.1	2.3E−05	BATDEDRAFT_34483		S
	Decreased	11.8	−0.9	1.9	2.8E−02	BATDEDRAFT_36120		S
	Decreased	10.4	−1.0	2.0	3.7E−03	BATDEDRAFT_5302, BATDEDRAFT_37484		S
	Decreased	13.0	−2.3	4.9	2.8E−03	BATDEDRAFT_36196		
**S41**								
	Increased	8.4	3.5	11.3	9.3E−06	BATDEDRAFT_85649		S
	Increased	8.7	2.8	7.0	3.2E−05	BATDEDRAFT_36653		Z
	Increased	9.2	1.6	3.0	3.5E−03	BATDEDRAFT_23534	*	
	Increased	9.4	2.7	6.5	5.2E−04	BATDEDRAFT_23310		
	Increased	9.0	2.5	5.7	7.3E−04	BATDEDRAFT_23544	*	
	Increased	8.8	2.3	4.9	4.1E−05	BATDEDRAFT_24208, BATDEDRAFT_24207		Z
	Increased	9.5	2.3	4.9	3.7E−05	BATDEDRAFT_24156		
	Increased	8.4	1.7	3.2	1.5E−03	BATDEDRAFT_87928	*	
	Increased	8.6	2.6	6.1	5.8E−06	BATDEDRAFT_24985	*	Z
	Increased	8.7	4.0	16.0	7.0E−05	BATDEDRAFT_25462	*	
	Increased	9.5	3.9	14.9	8.0E−06	BATDEDRAFT_35365		
	Increased	8.0	2.2	4.6	1.7E−02	BATDEDRAFT_90146		S
	Increased	8.6	0.8	1.7	2.3E−02	BATDEDRAFT_26287	*	
	Increased	8.6	3.5	11.3	5.5E−08	BATDEDRAFT_27937		S
	Increased	8.3	3.0	8.0	7.3E−07	BATDEDRAFT_92476	*	
	Decreased	9.9	−1.7	3.2	2.5E−03	BATDEDRAFT_86314		S
	Decreased	11.9	−1.1	2.1	6.5E−03	BATDEDRAFT_31623		S
	Decreased	13.2	−2.0	4.0	2.8E−02	BATDEDRAFT_37569		S
**ASP**								
	Increased	8.3	3.6	12.1	7.5E−08	BATDEDRAFT_21660	*	
	Increased	8.2	3.4	10.6	1.6E−06	BATDEDRAFT_22611	*	
	Increased	8.9	2.4	5.3	9.5E−06	BATDEDRAFT_22623	*	Z
	Increased	8.1	3.1	8.6	6.9E−06	BATDEDRAFT_86720	*	
	Increased	8.1	2.4	5.3	9.2E−06	BATDEDRAFT_23192	*	
	Increased	8.8	4.1	17.1	1.3E−07	BATDEDRAFT_23275	*	
	Increased	8.5	3.5	11.3	1.9E−07	BATDEDRAFT_87177	*	Z
	Increased	8.9	4.5	22.6	7.5E−08	BATDEDRAFT_87185	*	
	Increased	8.4	3.2	9.2	4.2E−06	BATDEDRAFT_87250	*	
	Increased	8.3	3.8	13.9	5.2E−08	BATDEDRAFT_23765	*	
	Increased	8.1	2.9	7.5	2.7E−06	BATDEDRAFT_87859	*	
	Increased	8.4	2.8	7.0	8.8E−06	BATDEDRAFT_87892	*	
	Increased	8.5	2.7	6.5	5.0E−06	BATDEDRAFT_88273	*	
	Increased	7.7	2.6	6.1	4.6E−04	BATDEDRAFT_24300	*	
	Increased	8.8	3.0	8.0	9.3E−06	BATDEDRAFT_24380	*	
	Increased	8.3	3.5	11.3	3.9E−06	BATDEDRAFT_24760	*	
	Increased	7.9	3.0	8.0	1.3E−03	BATDEDRAFT_24767	*	
	Increased	8.5	2.5	5.7	3.0E−04	BATDEDRAFT_25148	*	
	Increased	8.3	3.1	8.6	3.3E−07	BATDEDRAFT_25666	*	
	Increased	8.3	2.5	5.7	1.1E−03	BATDEDRAFT_36999		
	Increased	8.4	3.5	11.3	5.7E−07	BATDEDRAFT_89345	*	
	Increased	8.1	2.9	7.5	3.2E−05	BATDEDRAFT_25259	*	
	Increased	8.2	3.3	9.8	1.5E−06	BATDEDRAFT_89380	*	
	Increased	7.8	2.8	7.0	1.6E−06	BATDEDRAFT_25355	*	
	Increased	8.5	3.4	10.6	7.5E−08	BATDEDRAFT_89821	*	
	Increased	8.4	4.5	22.6	4.0E−06	BATDEDRAFT_25680		
	Increased	8.3	2.6	6.1	4.4E−05	BATDEDRAFT_25784	*	
	Increased	7.8	2.9	7.5	1.1E−06	BATDEDRAFT_89959	*	
	Increased	7.8	2.7	6.5	4.8E−03	BATDEDRAFT_26088	*	
	Increased	7.8	2.9	7.5	4.8E−04	BATDEDRAFT_26151	*	
	Increased	8.6	3.3	9.8	3.6E−06	BATDEDRAFT_26132	*	
	Increased	9.0	5.7	52.0	9.8E−07	BATDEDRAFT_90236	*	
	Increased	9.5	4.8	27.9	1.5E−04	BATDEDRAFT_90237	*	
	Increased	7.8	2.5	5.7	9.6E−05	BATDEDRAFT_26134	*	
	Increased	8.5	3.5	11.3	1.3E−06	BATDEDRAFT_90267	*	
	Increased	8.2	3.5	11.3	1.2E−06	BATDEDRAFT_26425	*	
	Increased	8.3	3.6	12.1	1.8E−06	BATDEDRAFT_90625	*	
	Increased	8.0	2.9	7.5	2.3E−06	BATDEDRAFT_26741	*	
	Increased	9.4	2.3	4.9	4.9E−06	BATDEDRAFT_35725	*	
	Increased	9.2	2.1	4.3	6.2E−04	BATDEDRAFT_90888	*	
	Increased	7.8	2.7	6.5	5.1E−05	BATDEDRAFT_26762	*	
	Increased	9.0	3.8	13.9	1.2E−07	BATDEDRAFT_26758	*	
	Increased	8.8	2.3	4.9	3.5E−03	BATDEDRAFT_26748	*	
	Increased	8.7	3.9	14.9	1.3E−06	BATDEDRAFT_90978	*	
	Increased	9.1	1.3	2.5	4.7E−04	BATDEDRAFT_91075		
	Increased	8.0	2.8	7.0	2.9E−06	BATDEDRAFT_27277	*	
	Increased	8.3	3.6	12.1	7.5E−08	BATDEDRAFT_27069	*	
	Increased	8.3	2.5	5.7	3.5E−03	BATDEDRAFT_27286		
	Increased	7.5	2.0	4.0	1.7E−02	BATDEDRAFT_27286		
	Increased	8.4	4.0	16.0	6.0E−06	BATDEDRAFT_27300	*	
	Increased	8.4	3.3	9.8	1.0E−05	BATDEDRAFT_92020	*	
	Increased	7.9	2.5	5.7	7.5E−05	BATDEDRAFT_27966	*	
	Increased	8.3	3.7	13.0	6.0E−08	BATDEDRAFT_28166		
	Increased	8.2	2.6	6.1	6.2E−05	BATDEDRAFT_28328	*	
	Increased	9.1	3.4	10.6	2.1E−06	BATDEDRAFT_28256	*	
	Increased	8.3	2.7	6.5	7.0E−06	BATDEDRAFT_92488	*	Z
	Increased	10.1	1.1	2.1	1.1E−02	BATDEDRAFT_92592	*	S
	Increased	7.9	2.8	7.0	1.5E−07	BATDEDRAFT_28513	*	
	Increased	8.7	3.9	14.9	5.5E−05	BATDEDRAFT_92740		
	Increased	8.4	3.4	10.6	1.3E−07	BATDEDRAFT_28541	*	
	Increased	7.5	1.5	2.8	5.1E−03	BATDEDRAFT_28537	*	
	Increased	8.3	3.3	9.8	5.5E−06	BATDEDRAFT_28684	*	
	Decreased	10.9	−1.3	2.5	4.1E−03	BATDEDRAFT_22179		S
	Decreased	11.3	−1.9	3.7	6.2E−05	BATDEDRAFT_9451		Z
	Decreased	13.3	−1.7	3.2	1.1E−02	BATDEDRAFT_16209		
	Decreased	11.2	−1.3	2.5	8.1E−03	BATDEDRAFT_87455, BATDEDRAFT_87454		S
	Decreased	11.9	−1.0	2.0	2.2E−02	BATDEDRAFT_37184		S
	Decreased	12.9	−2.1	4.3	8.0E−04	BATDEDRAFT_35816		Z
	Decreased	12.4	−2.1	4.3	2.7E−06	BATDEDRAFT_4465	*	Z
	Decreased	13.2	−1.1	2.1	9.7E−03	BATDEDRAFT_37356	*	
	Decreased	12.6	−1.1	2.1	1.9E−02	BATDEDRAFT_28984		S
**Adhesin**								
	Increased	9.0	1.9	3.7	2.0E−04	BATDEDRAFT_84886		
	Increased	9.7	2.8	7.0	8.7E−04	BATDEDRAFT_22355	*	S
	Increased	11.0	1.1	2.1	3.6E−02	BATDEDRAFT_21697	*	Z
	Increased	8.8	1.8	3.5	9.8E−04	BATDEDRAFT_85835		
	Increased	10.9	1.4	2.6	1.6E−02	BATDEDRAFT_85893		S
	Increased	10.4	1.2	2.3	8.6E−04	BATDEDRAFT_22768	*	
	Increased	10.1	1.1	2.1	3.2E−02	BATDEDRAFT_87248	*	
	Increased	9.7	1.3	2.5	4.4E−03	BATDEDRAFT_87422	*	S
	Increased	8.1	2.7	6.5	4.9E−04	BATDEDRAFT_23878	*	Z
	Increased	10.9	1.0	2.0	6.3E−03	BATDEDRAFT_87864		S
	Increased	8.9	2.9	7.5	9.7E−06	BATDEDRAFT_87929	*	
	Increased	8.6	3.4	10.6	3.9E−06	BATDEDRAFT_11071	*	
	Increased	8.1	2.9	7.5	1.1E−06	BATDEDRAFT_90520	*	
	Increased	9.3	2.8	7.0	5.4E−05	BATDEDRAFT_27092		
	Increased	8.2	3.2	9.2	1.0E−06	BATDEDRAFT_27091	*	
	Increased	8.7	2.4	5.3	2.3E−05	BATDEDRAFT_28554	*	
	Increased	8.5	3.5	11.3	7.5E−08	BATDEDRAFT_93124		
	Decreased	10.7	−1.6	3.0	3.7E−05	BATDEDRAFT_84904		S
	Decreased	10.8	−2.0	4.0	2.3E−03	BATDEDRAFT_34648		S
	Decreased	13.2	−0.9	1.9	1.8E−02	BATDEDRAFT_35182		
	Decreased	11.5	−2.5	5.7	4.1E−05	BATDEDRAFT_91430		S
**CRN**								
	Increased	10.3	1.4	2.6	4.5E−03	BATDEDRAFT_84882	*	
	Increased	10.6	1.3	2.5	2.4E−03	BATDEDRAFT_85109	*	
	Increased	7.9	2.8	7.0	3.4E−05	BATDEDRAFT_86517	*	
	Increased	9.0	2.1	4.3	9.3E−03	BATDEDRAFT_31422	*	Z
	Increased	8.0	2.2	4.6	1.4E−02	BATDEDRAFT_23217	*	
	Increased	11.2	4.3	19.7	5.5E−05	BATDEDRAFT_87221	*	Z
	Increased	9.4	2.0	4.0	5.7E−03	BATDEDRAFT_87524	*	Z
	Increased	8.9	3.0	8.0	2.4E−05	BATDEDRAFT_24811	*	Z
	Increased	8.6	1.2	2.3	3.1E−02	BATDEDRAFT_37012	*	Z
	Increased	8.4	2.5	5.7	4.7E−03	BATDEDRAFT_26085	*	Z
	Increased	8.8	1.9	3.7	4.2E−03	BATDEDRAFT_26137	*	Z
	Increased	9.3	1.7	3.2	4.5E−03	BATDEDRAFT_90343	*	Z
	Increased	8.9	1.6	3.0	6.2E−03	BATDEDRAFT_90726	*	Z
	Increased	8.0	3.0	8.0	1.5E−03	BATDEDRAFT_26749	*	Z
	Increased	8.4	2.5	5.7	4.3E−04	BATDEDRAFT_28183	*	Z
	Decreased	12.3	−1.7	3.2	7.6E−04	BATDEDRAFT_36061	*	Z
**Lipase 3**								
	Increased	8.5	3.6	12.1	1.4E−07	BATDEDRAFT_86691	*	Z
	Increased	9.1	2.2	4.6	8.4E−04	BATDEDRAFT_86693		
	Increased	9.0	2.7	6.5	2.6E−05	BATDEDRAFT_89307	*	
	Increased	9.0	1.8	3.5	8.7E−05	BATDEDRAFT_26489	*	Z
	Increased	8.5	3.8	13.9	1.5E−06	BATDEDRAFT_26490	*	
	Increased	8.3	1.8	3.5	1.7E−03	BATDEDRAFT_26491		
	Increased	8.5	3.0	8.0	1.1E−06	BATDEDRAFT_93190	*	
	Increased	8.6	3.6	12.1	4.8E−06	BATDEDRAFT_93191	*	

The “direction” column indicates whether genes showed increased or decreased expression in the frog skin treatment (relative to the tryptone treatment). Both log2 fold change and absolute-value fold change are given. The *p*-values are Benjamini and Hochberg corrected for multiple testing. The “Bd-specific” column indicates with asterisks those genes that were found to be unique to Bd [31]. The “life stage” column indicates those genes that were found to have significantly increased expression in the zoospore (Z) or sporangia (S) life stage [30].

In addition to the proteases gene families, we also found differential expression in several other gene families that have been hypothesized to play a role in Bd pathogenicity. First, a large family of Bd-specific crinkler like proteins (CRN) showed consistent patterns of increased expression when Bd was grown in frog skin (Fig. 1, Table 2). Second, a large number of adhesin genes were differentially expressed, primarily with increased expression in the frog skin treatment (Fig 1, Table 2). Notably, at least two of the adhesin genes with increased expression showed length variation with multiple alleles of different lengths. For the gene BATDEDRAFT_21697 we observed a 96 bp difference in length between the short and long alleles, and for the gene BATDEDRAFT_27091 we observed a 243 bp difference in length between the short and long alleles. Third, all of the Bd-specific lipases identified in Joneson et al. [31] showed significantly increased expression in frog skin (Table 2).

We also performed a second independent experiment using frog skin from a different species to determine whether our results were more general across substrates. The results from the two experiments were highly concordant with a correlation coefficient of 0.84 (p<<0.001). Specifically, 63.3% of the transcripts showed the same expression pattern (significant increase in expression, significant decrease in expression, or no change) across the two experiments. Only 1.2% of the transcripts showed conflicting expression patterns (significant increase in one experiment and significant decrease in the other). The remaining 35.5% of the transcripts were not informative for this analysis (no significant change in one experiment).

## Discussion

We evaluated the genomic signature of Bd during the infection of host tissue. Specifically, we measured gene expression across the genome of Bd on contrasting substrates (i.e., sterilized, pulverized amphibian skin *vs.* standard laboratory growth media). Our results indicate that nutrient conditions have an enormous impact on Bd gene expression. More than half of the genes in the Bd genome showed differential expression in different nutrient conditions. Many of the genes with increased expression in the standard growth media treatment (tryptone nutrient broth) were involved in basic cellular processes, possibly reflecting cellular activity at high growth rates. The specific genes with increased expression in the host tissue treatment provide insight into Bd pathogenicity at the molecular level and are the focus of our discussion here.

The evidence for the role of secreted proteases as Bd pathogenicity factors is gaining strength. Specifically, there are three protease gene families that merit consideration as Bd pathogenicity factors: metallo-, serine-, and aspartyl-proteases. The Bd genome contains gene family expansions for each of these gene families; M36, S41, and ASP genes are found in high copy number relative to other fungal genomes [31]–[32]. In previous work, we demonstrated that these gene family expansions are recent and have occurred specifically along the evolutionary branch leading to Bd [32]. By comparing the genome of Bd to that of a close non-pathogenic relative we showed that some of these protease paralogs are “Bd-specific” (i.e., recent duplicates found only in Bd and not other sequenced chytrids, [32]). In previous work we also demonstrated that some of these gene family members show life stage specific patterns of gene expression that are consistent with a role in the infection process [31].

Here we add a critical functional finding to the protease story by demonstrating that a large proportion of serine-, metallo-, and aspartyl-protease gene family members show increased expression when Bd is grown in host tissue (Fig. 1). When only the “Bd-specific” protease gene set is considered, the pattern is even more striking: 85% of the Bd-specific protease exhibit increased expression while only 3% show decreased expression. The fact that the vast majority of these protease genes show a consistent substrate-specific expression pattern suggests their interaction with host tissue. Combining data from this study with two of our previous studies [31]–[32], we can identify specific protease genes that represent particularly important targets for future study. In Table 2, we highlight a number of genes that show increased expression in host tissue, are Bd-specific, and show life stage specific patterns of expression. Functional studies of the secreted proteases encoded by these genes could provide insight into the initial invasion of host cells and the metabolism of host tissue during the establishment of infection.

In addition to the secreted proteases, we documented several other categories of Bd genes with consistently increased expression in host nutrient conditions. Here we highlight several particularly intriguing groups. First, we were interested in the potential role of adhesin genes in Bd pathogenicity. The protein composition of the fungal cell wall is thought to be dynamic, changing in part based on the substrates encountered [36]. Important components of the cell wall are adhesin proteins that allow fungal cells to adhere to both self and non-self [37]–[38]. In some pathogenic fungi, adhesin proteins are thought to play a role in pathogenicity by facilitating adhesion to host cells and evasion of host detection [39]. Specifically, adhesion proteins can contain regions of variable repeats, and protein length variation can help pathogenic fungi evade cell surface recognition by the host [39]. We identified 11 adhesin genes in Bd with increased expression in the frog-skin treatment, and almost all of these were Bd-specific. Notably, we found that at least two of these adhesins show length variation, with different length variants in different isolates and occasionally multiple length variants in a single isolate. Because adhesin allele length variation has been correlated with differences in adhesive strength in other fungal pathogens [39]–[41], future studies could investigate the functional consequences of observed adhesin allele length variation in Bd. Future studies could also characterize the distribution of allelic variation in adhesin genes across a larger sample of Bd isolates.

Second, we were interested in a large group of Bd-specific genes that show sequence similarity to Crinklers and Crinkler-like effectors (CRN). These genes have never been found in other fungi, but have been reported from oomycetes, a group of Chromista pathogens [42]–[43]. Recently Sun et al. [44] suggested that Bd’s CRN genes may have been acquired by lateral transfer, but this hypothesis needs to be rigorously evaluated in a phylogenetic context. The Bd Crinkler and CRN genes showed a strong and consistent signal of increased expression in the frog skin treatment. Genes in this functional group were also more highly expressed in the zoospore life stage compared with the sporangia life stage. This is particularly interesting because the signal is highly consistent (13 of 16 genes) and because relatively few genes in the Bd genome have increased expression in the zoospore stage [31]. Our functional data therefore suggest that CRN genes merit detailed investigation in Bd.

Third, we were interested in a group of triglyceride lipases. These genes have lipase-3 protein domains and many of them were found to be Bd-specific in a previous study [32]. Here we show that these genes all show significantly increased expression in the frog skin treatment, often with very large fold changes. A specific role for lipases in Bd pathogenesis has not yet been proposed. However, lipases are involved in other fungal-host interactions and in at least one fungal-vertebrate interaction [45]–[46]. Lipase activity has not been evaluated in most of the studies that have investigated Bd enzymatic activity (e.g., [24], [47]). However Symonds et al. [48] showed a weak reaction of Bd in the presence of esterase lipase (C8) and lipase (C14). The increased expression of lipases in our frog skin treatment could also be due to the increased availability of lipases in pulverized tissue (i.e. from subcutaneous adipose cells that would not typically be available to Bd in live hosts). Therefore future tests of Bd enzymatic activity should explicitly test whether Bd lipases are activated in this way in intact amphibian skin.

Our results were robust across replicated experiments, which used different Bd isolates and skin substrate from different hosts. Future studies could extend our work in several fruitful ways. First, our study was not designed to evaluate potential differences in gene expression among isolates. Isolates that vary in virulence could be explicitly compared to determine whether different isolates exhibit predictable difference in gene expression profiles during infection. This could be extremely important if, for example, particular genes are induced only in especially virulent isolates. Second, isolates grown on different host species substrates could be explicitly compared to determine whether there are genes that are induced in a host-specific manner. Gene expression studies in conjunction with histological examination could help determine if there is something fundamentally different about skin “invisibility” in Bd-resistant amphibians. Third, further study of Bd gene expression *in vivo* - either in the lab or in the wild - will be important to fully evaluate the genetics of the interaction between Bd and its amphibian hosts. There may be Bd genes induced only when Bd infects *live hosts* or in response to specific physiological and immunological host activities, which would not be captured using *in vitro* methods. Further, our use of pulverized skin tissue (where all nutrients may be available immediately to Bd), may simplify what is a temporally dynamic process *in vivo*. Finally, a focus on host cellular processes will be necessary to determine what specific host proteins Bd is responding to and interacting with. Therefore, it will be important to assay Bd gene expression directly from infected host tissue to evaluate the induction of Bd genes in the natural context of infection.

## Materials and Methods

We conducted a replicated *in vitro* global gene expression experiment to compare Bd gene expression in standard growth media versus sterilized frog skin.

### Chytrid Culturing

We used six independent Bd isolates as biological replicates. Four *Bd* isolates were from natural populations of *Rana muscosa* and *Rana sierrae* in California (JAM81, JAM88, TST75, and TST77), one *Bd* isolate was from a natural population of *Phyllomedusa lemur* from Panama (JEL423), and one *Bd* isolate was from a natural population of *Batrachoceps attenuatus* from California (SW11). The isolates selected were from the more recently derived, globally distributed Bd clade (termed the Global Panzootic Lineage (“GPL”) by [49]). It is important to note that we designed our study to compare Bd gene expression in different nutrient conditions, rather than to examine molecular differences among isolates as has been done in other studies (e.g., [50]). Therefore we maximized the number of isolates rather than the number of replicates of each isolate. Each isolate was grown on 1% tryptone plates for 2 weeks at room temperature, and live zoospores were harvested by flooding the plates with sterile deionized (DI) water. Zoospores were washed 3 times with sterile DI water to ensure that no tryptone media was transferred into the experimental treatment flasks.

Each Bd isolate was then grown under two nutrient conditions: 1% liquid tryptone and 1% pulverized frog skin. The 1% tryptone broth was prepared with DI water, aliquoted, and autoclaved before inoculation with Bd. The preparation of the 1% frog skin broth was more involved to ensure that frog skin proteins remained intact and to eliminate microbial contaminants. We collected ventral and leg skin from adult cane-toads (*Bufo marinus*), a species that is fairly resistant to Bd. We flash-froze the samples and ground the skin in liquid nitrogen with a mortar and pestle. We did not autoclave the skin so as not to denature host proteins. Instead we sterilized the skin by submerging in 95% ethanol for 10 minutes, washed the skin 5 times in sterile DI water, submerged skin in 10% hydrogen peroxide, and washed the skin 5 more times in sterile DI water. We then made our final 1% solution of frog skin in DI water and subjected the solution to UV radiation for 1 hour.

We used a 250 mL flask containing 100 mL nutrient broth for each replicate. Each flask was inoculated with approximately 5 × 10^6^ zoospores. Flasks were incubated for 2 weeks at room temperature under agitation. At the end of the experimental period, a sample from each flask was checked for live Bd under magnification. Samples were then washed 3 times with sterile DI water, pelleted, frozen in RNAlater buffer (Ambion, Life Technologies, Grand Island, NY), and stored at −80C. The pellets contained the entire population of Bd present at the sampling point including all life stages.

### Molecular Methods

We designed a NimbleGen 12-plex microarray for *Bd* based on the publically available Bd genomes JAM81 (*B. dendrobatidis* Sequencing Project, Joint Genome Institute: http://genome.jgi-psf.org/Batde5/Batde5.download.ftp.html) and JEL423 (*B. dendrobatidis* Sequencing Project, Broad Institute of Harvard and MIT: http://www.broadinstitute.org/annotation/genome/batrachochytrium_dendrobatidis). The microarray contained 133,254 60-mer probes representing a possible 7,949 expressed transcripts (probe sets). Probe sets were defined as all probes that mapped to a single transcript, which in turn mapped to a single gene. There was an average of 20 probes per probe-set. RNA was isolated with Trizol/Chloroform (Invitrogen, Carlsbad, CA) with a standard protocol and DNase digestion (Ambion Turbo DNA-free DNase, Austin, TX). Double-stranded cDNAs were synthesized using Invitrogen’s SuperScript cDNA Synthesis Kit with the standard protocol using oligo dT primers (Invitrogen, Carlsbad, CA). cDNAs were fluorescently labeled with Cy5 from TRILink BioTechnologies (TRILink BioTechnologies, San Diego, CA) using the standard NimbleGen Gene Expression Analysis v3.2 protocol.

All samples were hybridized to a single 12-plex chip to reduce technical noise. An 18-hour hybridization was conducted at 42 degrees Celsius in a NimbleGen Hybridization System 4 chamber (NimbleGen, Madison, WI). The chip was washed in an automated MAUI Wash System (BioMicro Systems, Salt Lake City, UT) and then scanned on an Axon GenePix 4000B Scanner (Molecular Devices, Sunnyvale, CA) using GenePix Pro v6.1 software (Molecular Devices, Sunnyvale, CA).

### Data Analysis

NimbleScan v2.5 software (NimbleGen. Madison, WI) was used to align a chip-specific grid to control features and extract raw intensity data for each probe and each array. Chip images were then visually checked for each array and verified not to contain any significant spatial artifacts. Raw intensity data was then read into the R statistical computing environment (www.R-project.org) and checked for quality. Further, chip intensity distributions, boxplots, and hierarchical clusters were compared and checked for any unusual global patterns. Each array was then background corrected and normalized using the quantile normalization procedure [48]. Finally each probe set was summarized using the median polish procedure as described with the robust multichip average (RMA) procedure [51]–[52]. The median polish procedure is a robust method for summarizing all probes contained within each probe set to a single expression value for each gene taking into account individual probe effects. Probe sets with particularly low (Interquartile Range, IQR <0.5) or particularly high (IQR>1.0) levels of expression variation across all samples were removed from further analysis, reducing the overall number of statistical tests to be performed. A total of 7,019 expressed transcripts remained after IQR filtering.

Differential expression was assessed using a linear model with an empirical Bayesian adjustment to the variances [53] and comparisons of interest were extracted using contrasts. The Benjamini and Hochberg (BH) method was used to control for the expected false discovery rate given multiple tests [54]. Probe sets were considered statistically differentially expressed with a BH adjusted p-value of <0.05. We report the log2 fold change values for all differentially expressed probe sets. All microarray data is publicly available in accordance with MIAME (Study accession: GSE37135; Bd custom platform accession GPL15422).

For Bd gene annotation, we used Gene Ontology (GO) terms, Protein family (Pfam) domains and InterPro signatures. Of the 7,019 expressed transcripts analyzed, 4,655 could be confidently annotated. We performed Gene Set Enrichment Analysis (GSEA) to test for significant enrichment of particular Gene Ontology terms [55]. The purpose of GSEA is to incorporate biological knowledge and identify gene sets (genes grouped based on a common function or pathway) with enriched expression in one of the treatment groups (frog skin vs. nutrient broth). We conducted GSEA separately for the entire collection of Bd genes represented on our microarray and on a subset of “Bd-specific” genes. We evaluated Bd-specific genes because they are of particular evolutionary interest; these genes have undergone recent evolution or duplication in Bd because they do not have clear orthologs in a close non-pathogenic relative [32].

We also looked at particular functional groups (genes with specific GO, Pfam or InterPro terms) that were of *a priori* interest as putative Bd pathogenicity factors. We focused on three families of proteases (serine, metallo, and aspartyl), which show lineage-specific gene family expansions in Bd [32]. We also evaluated patterns of expression for two additional gene families that are largely Bd specific and may be involved in adhesion (i.e., adhesins) or pathogenesis (i.e., CRN). Because length-variation in adhesin genes has been shown to be associated with functional variation in fungal cell adhesion [39]–[41], we also looked for length variation in adhesin alleles in a sample of Bd isolates. We identified putative adhesin genes (hereafter referred to as “adhesins”) in the Bd genome by searching for proteins with signal peptides (SP), GPI-anchors (using GPI-SOM [gpi.unibe.ch]), at least 2 repeat regions of 9 or more amino acids (using RADAR [56]), and that were heavily glycosylated (using YinOYang [57]). We found four putative adhesins that were predicted to show length variation based on the two sequenced Bd genomes (gene IDs: BATDEDRAFT_22355, BATDEDRAFT_21697, BATDEDRAFT_24031, BATDEDRAFT_27091). We designed PCR primers for these genes (primer sequences provided in Table S1) and amplified them in six Bd isolates (Isolate identifiers: CJB4, CJB5-2, CJB7, JEL289, JEL423, JEL627). We cloned and sequenced alleles for loci that showed more than one PCR product as visualized by gel electrophoresis.

### Replication

To test the generality of our results across different frog skin substrates, we conducted a small-scale replication of our entire experiment. For this replicate we used ventral and leg skin from adult African clawed frogs (*Silurana tropicalis*), a species that is fairly susceptible to Bd. For this replicate we used three Bd isolates (JAM81, JEL423, and TST75) as biological replicates. All molecular methods were repeated as described above, although we used a 1-plex array because of the smaller number of samples. We evaluated the correlation of expression pattern of all genes common across the two experiments. Specifically, we permuted the data 100,000 times and at each permutation calculated the correlation between the log2 fold change values in the two experiments. We calculated a permuted P-value as the number of permutation-based correlations greater than the test correlation divided by the number of permutations performed.

## Supporting Information

Table S1 **Primers for four Bf adhesin loci.** Primers do not all capture the complete predicted coding regions but do capture the region of length variation.
(DOCX)
