# Supplementary material for: Substrate-Specific Gene Expression in Batrachochytrium dendrobatidis, the Chytrid Pathogen of Amphibians
Source: PLoS One. 2012 Nov 20;7(11):e49924. doi: 10.1371/journal.pone.0049924 (PMC3502224; doi:10.1371/journal.pone.0049924)
Supplement: Table S1 — Primers for four Bf adhesin loci. Primers do not all capture the complete predicted coding regions but do capture the region of length variation. (DOCX) [file pone.0049924.s001.docx]

**Supporting Information Table S1:**

| Locus ID | Forward primer(s) | Reverse primer(s) |
| --- | --- | --- |
| BATDEDRAFT_22355 | F1: CTTGCTCTTGCCACTC  F2: AGGCACCAGCTAAACAGA | R1: GTCACTTTTACATTGCCACC |
| BATDEDRAFT_21697 | F1: GGGCTGATTGACCTATCTT  F2: ACTCCTGGAGATGATTCC | R1: CAAACACTCCATACATATCC |
| BATDEDRAFT_24031 | F1: CGCTCTTGTTGCTGCTAT | R1: CTATCCAGCGGCCATTTT |
| BATDEDRAFT_27091 | F1: GTCAAATACAGATTCAAGCTG  F2: TGCAGCCAATAATCCCATAC | R1: CTCATCCCTCTTATCACCA  R2: GCAATATCAGATTTTGACAAGTC |
